# Administration of an Intravenous Fat Emulsion Enriched with Medium-Chain Triglyceride/ω-3 Fatty Acids is Beneficial Towards Anti-Inflammatory Related Fatty Acid Profile in Preterm Neonates: A Randomized, Double-Blind Clinical Trial

**DOI:** 10.3390/nu12113526

**Published:** 2020-11-16

**Authors:** Panos Papandreou, Aristea Gioxari, Dimitrios Ntountaniotis, Olga-Natalia Korda, Maria Skouroliakou, Tania Siahanidou

**Affiliations:** 1 First Department of Pediatrics, Medical School, National & Kapodistrian University of Athens, “Aghia Sophia” Children’s Hospital, Goudi, 11527 Athens, Greece; ppapandreou@cibusmed.com (P.P.); siahan@med.uoa.gr (T.S.); 2 Department of Nutrition, IASO” Maternity Hospital, Marousi, 15123 Athens, Greece; 3 Department of Nutritional Science and Dietetics, Harokopio University, Kallithea, 17671 Athens, Greece; arisgiox@gmail.com (A.G.); olganataliakorda@gmail.com (O.-N.K.); 4 Department of Chemistry, National and Kapodistrian University of Athens, Zografou, 15771 Athens, Greece; doudaniotis@yahoo.gr

**Keywords:** parenteral nutrition, preterm, neonates, omega-3 fatty acids, fish oil, oleic acid, inflammation

## Abstract

Intravenous administration of pure soybean oil emulsions high in linoleic acid may lead to inflammation and lipid peroxidation in preterm neonates. We aimed to investigate the effects of a medium-chain triglyceride (MCT)/ω-3 polyunsaturated fatty acid (PUFA)-enriched intravenous fat emulsion (IVFE) on plasma fatty acid (FA) profile and serum interleukin-6 (IL-6) in preterm neonates. In this double-blind randomized study, 92 preterm neonates (gestational age < 32 weeks, birth weight < 1500 g) were assigned to receive either MCT/ω-3 PUFA-enriched IVFE (Intervention Group) or soybean oil-based IVFE (Control Group). Levels of FAs were measured at baseline (day 0) and day 15 of parenteral nutrition with gas-chromatography mass-spectrometry. Serum IL-6 was measured with sandwich ELISA in 59 neonates. Plasma FAs changed significantly over time; the MCT/ω-3 PUFA-IVFE group showed higher ω-3 PUFAs (*p* = 0.031), eicosapentaenoic acid (*p* = 0.000), and oleic acid (*p* = 0.003), and lower ω-6/ω-3 PUFAs ratio (*p* = 0.001) and ω-6 PUFAs (*p* = 0.023) compared to control group. Linoleic acid was higher in the soybean oil (SO)-based IVFE arm compared to the MCT/ω-3 PUFAs-IVFE arm (*p* = 0.006). Both fat emulsion types decreased IL-6 compared to baseline, but changes were insignificant between groups. Administration of MCT/ω-3 PUFA-enriched IVFE in preterm neonates is beneficial in changing the FA profile consistent with attenuated inflammatory response.

## 1. Introduction

Preterm neonates are in an energy-deficient state due to oxygen desaturation and hypoxia events, painful and stressful stimuli, illness, and rapid neurodevelopment [1,2]. Inadequate energy supply may be associated with impaired growth, increased severity of postnatal diseases, and adverse neurodevelopment [3,4,5]. Administration of emulsions enriched with fatty acids can provide high energy volumes of non-carbohydrate calories, thus avoiding osmotic overload [6,7,8]. Additionally, lipid emulsions contain high amounts of essential fatty acids, such as linoleic acid (LA) and alpha-linolenic acid (ALA) [9].

Following elongation and desaturation via a series of enzymatic reactions, LA and ALA can be converted to more physiologically active compounds, such as arachidonic acid (AA), eicosapentaenoic acid (EPA), and docosahexaenoic acid (DHA) [10]. However, the de novo synthesis rates are insufficient to maintain adequate plasma and erythrocyte concentrations of these long-chain polyunsaturated fatty acids (LC-PUFAs), indicating that DHA and AA should be considered conditionally as essential fatty acids for preterm neonates and should also be administered [11,12,13,14]. 

The LC-PUFAs (such as AA, EPA, and DHA) play a pivotal role in the prognosis of preterm neonates, affecting the development of the nervous system, the retina, and the visual cortex, as well as growth, body composition, immune allergic reactions, and the prevalence of chronic diseases in later life [15,16,17,18,19]. Most LC-PUFAs in the brain accumulate during the phase of rapid brain development. This phase takes place in the last trimester of pregnancy, and the first two years after birth [15,16,17,18,19]. Deteriorated nervous system development can lead to long-term negative effects beyond the period of nutritional deficiency. Therefore, it is essential to provide a source of LC-PUFAs to preterm infants, who lose this period of pregnancy [15,16,17,18,19]. 

Pure soybean oil (SO)-based intravenous fat emulsions (IVFEs) have been widely used for several decades in adults, children, and neonates. More recently, intravenous lipid emulsions were vegetable oil-based, until the newest ones available that contain fish oil [9]. The SO-IVFEs have high concentrations of essential fatty acids with a ratio of LA to ALA of approximately 8:1, but they lack appreciable amounts of other LC-PUFAs [20]. What is more, lipid emulsions based on pure SO may result in excess formation of pro-inflammatory eicosanoids, which may limit the availability of LC-PUFAs for central nervous system development and immune function [20,21,22,23,24]. The conversion of LA to AA, results in the synthesis of eicosanoids. Some of these metabolic pathways yield prostaglandin endoperoxides G2 and H2, prostaglandin E2 (PGE2), thromboxane A2 (TXA2), leukotrienes, and related metabolites. These have inflammatory actions in their own right and regulate the production of other mediators including inflammatory cytokines [22,23,24]. 

On the other hand, fish oil-based IVFEs that are rich in omega-3 (ω-3) fatty acids (including EPA), may compete with AA in order (i) to produce eicosanoids with beneficial effects on inflammation and (ii) to suppress the production of PGE2 and TXA2. Omega-3 fatty acids also have been described to decrease the production of pro-inflammatory cytokines such as tumor necrosis factor-alpha (TNF-α), interleukin-1 beta (IL-1β), and interleukin-6 (IL-6) [24,25,26]. Furthermore, EPA, and to a lesser extent DHA, follow anti-inflammatory metabolic paths that lead to the production of eicosanoids like prostaglandin E3 (PGE3), leukotriene B5 (LTB5), and thromboxane A3 (TXA3).

Therefore, the aim of the present study was to investigate the effects of administration of a medium-chain triglyceride (MCT)/ω-3 PUFA-enriched IVFE versus a soybean-based IVFE on plasma fatty acid profile, in preterm neonates.

## 2. Materials and Methods 

### 2.1. Study Design

**Figure 1:**
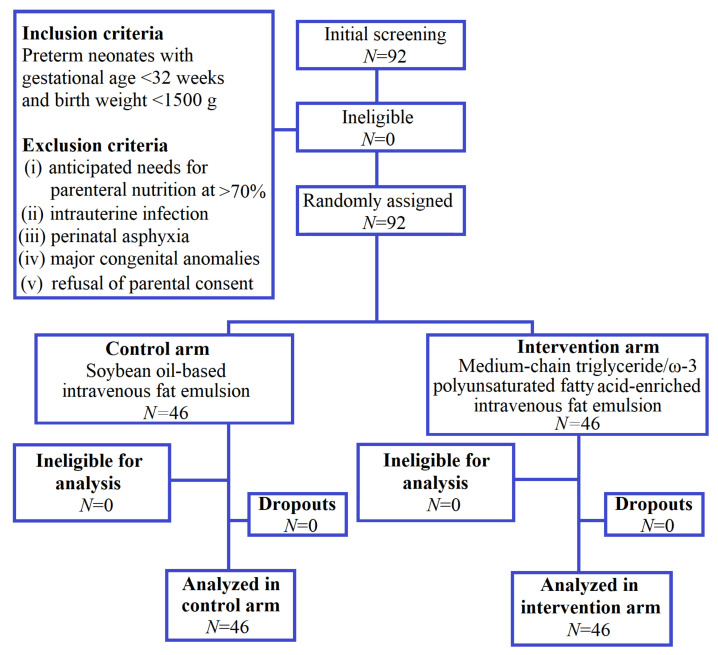
Study’s flowchart.

The study was performed as a randomized controlled double-blind clinical trial with parallel design (2 arms) and with a randomized allocation of 1:1 ratio between groups (Figure 1).

### 2.2. Participants 

Preterm neonates with gestational age <32 weeks and birth weight <1500 g that were admitted to a tertiary neonatal intensive care unit (between February 2017 and February 2018), within 12 h after birth, were assessed for eligibility. Exclusion criteria were anticipated needs for parenteral nutrition (PN) at >70% of total daily energy for <10 days, evidence of intrauterine infection, perinatal asphyxia, major congenital anomalies, and refusal of parental consent. 

### 2.3. Bioethics 

The study protocol was approved by the Scientific and Ethical Committee of (removed for blind review) (#355/15-2-18). The study was conducted in accordance with the Declaration of Helsinki. Neonates’ parents gave their informed consent for inclusion before they participated in the study. This work is registered in ClinicalTrials.gov (Protocol Record 201802). 

### 2.4. Intervention Procedures 

The preterm neonates were randomly allocated to one of the following two groups: the intervention group, which received an intravenous fat emulsion enriched with MCT/ω-3 PUFAs, and the control group, receiving a SO-based formula. Fat emulsion was added in the PN solution on the first or second day of life at a dose of 1 g/kg/day, which was increased by 1 g/kg/day up to a maximum amount of 3 g/kg/day. 

In the intervention group (MCT/ω-3 PUFA-IVFE group), the source of parenteral fat was Smoflipid (Fresenius Kabi HELLAS, Athens, Greece), a formulation containing MCTs, soybean oil, olive oil, fish oil, and alpha-tocopherol. The conventional soybean oil-based lipid formulation Intralipid 20% (Fresenius Kabi HELLAS, Athens, Greece) was administered to the control group (SO-IVFE group). Compositions of the two fat emulsions are presented in Table S1. Amino acids derived from Vamin Infant (Fresenius Kabi HELLAS, Athens, Greece) were administered in both groups. 

Enteral feedings were applied as soon as possible with either maternal milk or DHA-enriched preterm formula (PreNAN Discharge, Νestlé, HELLAS, Athens, Greece). The milk formula provides 73 kcal/100 mL of energy with carbohydrate-, protein-, and fat content of 7.7%, 2.0%, and 3.8%, respectively. Concentrations of LA, ALA, DHA, and a-tocopherol are 6.0 mg/mL, 752 ng/mL, and 144 ng/mL and 16 mg/L, respectively. 

Neonates were fed exclusively with PN for at least the first 3 days of life. Then, enteral feedings were provided in volumes that were determined by neonates’ weight and energy requirements on a daily basis. Parenteral solutions were administered to neonates (in both groups) until oral feedings reached a minimum of 80% of total energy intake. Eligibility, based on the inclusion and exclusion criteria, was assessed by the neonatologists of the neonatal intensive care unit.

### 2.5. Clinical Data

Before the start of the trial, the following data were collected: Gestational age, birth weight, perinatal history, and neonatal medical conditions and treatment. In addition, parenteral and enteral nutrition intakes, as well as changes in body weight, were recorded daily. Other parameters such as white blood cell and platelet counts, hematocrit, and C-reactive protein levels were assessed only if it was indicated for clinical reasons. Measurements were performed at baseline (day 0) and day 15 of PN. Blood samples were obtained after PN was temporally ceased for 4 h and before oral feeding. 

### 2.6. Fatty Acid Assessment with Gas Chromatography 

Plasma was separated from ethylenediaminetetraacetic acid treated blood samples after centrifugation at 1500 g for 15 min at 4 °C and were stored at −80 °C for further analyses. Initially, plasma samples were thawed and homogenized with stirring using Vortex. They were transferred to screw-on glass test tubes and then cooled to −80 °C and dehydrated by lyophilization. Fatty acid methyl esters were prepared by the method of Lepage G. and Roy C.C. (1986), modified by Rodríguez-Palmero et al. (1997) using acetyl chloride and methanol [27,28].

Agilent HP6890 gas chromatograph equipped with a Flame Ionization Detector (FID), MS 6890 Mass Selective Detector (MSD), and HP 7673 auto sampler was used. Methyl esters of the fatty acids were separated on a SGE BPX 70 capillary column, 60 m in length, 0.25 mm in diameter, and 0.25 μm in inner thickness. Helium was used as a carrier gas at a flow rate of 0.8 mL/min while the temperature of the sample imager and detector was 230 °C and 290 °C, respectively.

For the analysis, 1 µL of sample was injected into the split mode chromatograph at a ratio of 20:1. Thus, finally 1/20 of 1 µL was introduced into the column. The fatty acid retention times were initially recorded by analyzing 37 multi-standard fatty acid methyl esters of Sigma (Sigma L9405, Sigma-Aldrich, Seelze, Germany) using the MSD and with the help of the NIST and WILEY electronic libraries. The flame ionization detector was then used and the standards were intermixed at regular intervals to record any slight changes in the retention times of the constituents and to calculate the response factors (RFs) of the various fatty acids, so that the areas of the corresponding chromatographic peaks were corrected. The relative proportions of fatty acids (% of total fatty acids) were determined by the completion of chromatographic peaks and corrected with the use of RFs. 

### 2.7. Serum Interleukin-6 Measurement

Serum isolation was performed in a subset of the enrolled neonates (*n* = 59) due to evidence of hemolysis. Sera were separated from blood samples after centrifugation at 1800 g for 10 min at 4 °C and were stored at −80 °C for further analyses. 

Sandwich Enzyme Linked-Immunosorbent Assay (ELISA) kit was used to measure serum IL-6 levels (pg/mL) according to the manufacturer’s instructions (R&D Systems Inc., Abingdon, UK).

### 2.8. Randomization, Sequence Generation, and Implementation

The sample randomization was conducted by a computer-generated randomization list. A pharmacist received the list, prepared the different parenteral formulations in identical bags, and finally assigned each neonate to control or intervention group. The pharmacist was not involved in neonates’ care. All medical personnel and participants were blinded to treatment assignment during the whole study period.

### 2.9. Primary Outcomes and Sample Size

Our primary outcome was the detection of clinically significant differences in plasma ω-3 PUFAs, ω-6 PUFAs and EPA in the MCT/ω-3 PUFA-enriched IVFE group compared to the SO-IVFE group post-intervention. Based on previously published work of our research team [29], a minimum sample size of 14 neonates (7 per arm) was sufficient to result in a clinically important difference of 2 in plasma ω-3 PUFAs change between the control- [standard deviation of mean (SD) = 1] and the MCT/ω-3 PUFAs arm (SD = 1.5) using a two-tailed t test with 80% power and a 5% level of significance. Similarly, to detect a significant difference of 2 in total ω-6 PUFAs, 50 neonates (25 per arm) were needed (control: SD = 2.5, intervention: SD = 2.5). Additionally, a significant difference of 0.5 in EPA required 20 neonates per arm (control: SD = 0.5, intervention: SD = 0.6). Secondary outcomes of our study were significant changes in serum IL-6, plasma ω-6/ω-3 PUFAs ratio, and levels of linoleic acid, DHA, and oleic acid. A minimum sample size of 318 neonates (159 per arm) was needed to observe a significant difference of 3.5 in serum IL-6 concentration between the two groups post-intervention (control: SD = 13, intervention: SD = 11.5).

### 2.10. Statistical Analysis 

All analyses were conducted by applying the Statistical Package for the Social Sciences (SPSS 21.0 for Windows, Chicago, IL, USA). Descriptive statistics were calculated for all parameters and the Kolmogorov–Smirnov test was applied to investigate if all measures were characterized by normal distribution. Parametric data are expressed as mean values (±SD), while non parametric data are expressed as medians and interquartile ranges. For variables with normal distribution, the independent samples t-test was applied to compare the differences between the two arms pre- and post-intervention, while for variables without normal distribution, the Mann–Whitney test was applied. Before the intervention, this test served to ensure that the study population was characterized by homogeneity. For investigating possible intra-group differences, a paired samples t-test was applied for parametric variables and the Wilcoxon test for non-parametric ones. For all statistical analyses, significance was set at *p* < 0.05.

## 3. Results

A total of 92 preterm neonates were included and completed the study: 46 in the control and 46 in the intervention group. Clinical characteristics of each study group at baseline are shown in Table 1. No statistically significant differences between the two groups were observed for gestational age (*p* = 0.126), birth weight (*p* = 0.995), serum IL-6 concentration (*p* = 0.383), and plasma fatty acid (FA) profile. 

Body weight increased significantly in both the control (1331.30 ± 237.56 g vs. 1223.04 ± 215.42 g, *p* = 0.000) and the intervention arm (1339.05 ± 211.02 g vs. 1222.74 ± 211.90 g, *p* = 0.000) at day 15 compared to baseline. The increase in body weight did not differ between groups (*p* = 0.722). Safety and tolerability: both IVFEs were well tolerated. Serum triglyceride levels were within normal values for age, whereas no local reaction, thrombocytopenia that could be attributed to IVFE, or dropout related to any IVFE-associated adverse effect were observed.

### 3.1. Dietary Intake

The energy intake via parenteral nutrition, as well as the amount of enteral feeding of preterm neonates in the control (SO-IVFE) and the intervention (MCT/ω-3 PUFAs-IVFE) group, are presented in Table 2. No significant differences were observed at baseline and at the endpoint of the study between the two groups. At baseline, no neonate received enteral feeding (maternal milk or formula). Nutrient and mineral intake via enteral and parenteral routes did not differ at day 15 between the two arms (Table S2). 

### 3.2. Plasma Fatty Acids

Plasma fatty acid profiles of the two study arms pre- and post-intervention are presented in Table 3. Parenteral nutrition significantly increased levels of total PUFAs at day 15 compared to baseline, both in the control (*p* = 0.000) and the intervention arm (*p* = 0.000). However, the change in total PUFAs did not differ between groups post-intervention (*p* = 0.099). 

### 3.3. Polyunsaturated Fatty Acids

In regards to plasma total ω-6 PUFAs, both groups showed significant elevations at day 15 compared to day 0, but the raise was significantly lower in the MCT/ω-3 PUFAs group than the SO group (*p* = 0.023). Augmentation of total ω-3 PUFAs in the intervention arm did not reach statistical significance (*p* = 0.070), but post-intervention, the mean difference between arms was significant (*p* = 0.031). In the control arm, total ω-3 PUFAs did not change throughout the trial (*p* = 0.131). The ω-6/ω-3 PUFAs ratio was not altered in the intervention group (*p* = 0.202), but it was significantly increased in the SO group (*p* = 0.000). This change remained significant when compared to the MCT/ω-3 PUFAs group post-intervention (*p* = 0.001).

Among ω-6 PUFAs, levels of LA (C18:2ω-6) were significantly increased in both the SO group (*p* = 0.000) and the intervention group (*p* = 0.000) compared to baseline, but the increment was more profound in the control than the intervention group (*p* = 0.006). Concentration of γ-linolenic acid (GLA) (C18:3ω-6) was raised only in the SO arm (*p* = 0.040), but this increase was not significant when compared to the MCT/ω-3 PUFAs arm post-intervention (*p* = 0.231). At day 15, AA (C20:4ω-6) concentration was significantly decreased in both the control (*p* = 0.000) and intervention groups (*p* = 0.000) compared to baseline, but changes between groups were found insignificant (*p* = 0.204). 

Regarding ω-3 PUFAs, EPA (C20:5ω-3) rise was higher in the MCT/ω-3 PUFAs than the SO group (*p* = 0.000), while DHA (C22:6ω-3) dropped in both study arms (control arm: *p* = 0.000; intervention arm: *p* = 0.001). Mean changes of DHA levels were found to be insignificant between groups (*p* = 0.204). Additionally, levels of ALA (C18:3ω-3) were elevated in both SO (*p* = 0.000) and MCT/ω-3 PUFAs arms (*p* = 0.030), however the increase was significantly higher in the control than the intervention group (*p* = 0.006). 

### 3.4. Monounsaturated Fatty Acids

Total MUFAs did not change throughout the study (control arm: *p* = 0.390; intervention arm: *p* = 0.575). Nevertheless, oleic acid (OA) (C18:1ω-9) was significantly elevated in the intervention arm (*p* = 0.000) but not in the control arm (*p* = 0.933) at day 15 compared to day 0, and this increase remained significant when compared to control group post-intervention (*p* = 0.003).

### 3.5. Saturated Fatty Acids

Levels of SFAs dropped in both SO (*p* = 0.000) and MCT/ω-3 PUFAs group (*p* = 0.000) compared to baseline, and changes between groups post-intervention were insignificant (*p* = 0.349). Significant reductions were observed for the following SFAs in both groups: Pentadecylic acid (C15:0) (control arm: *p* = 0.000; intervention arm: *p* = 0.000), palmitic acid (C16:0) (control arm: *p* = 0.000; intervention arm: *p* = 0.000), margaric acid (C17:0) (control arm: *p* = 0.000; intervention arm: *p* = 0.000), and lignoceric acid (C24:0) (control arm: *p* = 0.001; intervention arm: *p* = 0.000). Myristic acid (C14:0) exhibited a significant increase in both SO and MCT/ω-3 PUFAs arm (control arm: *p* = 0.000; intervention arm: *p* = 0.001) but changes were not significant between groups (*p* = 0.128).

### 3.6. Serum IL-6 Levels 

Both fat emulsion types managed to decrease serum IL-6 levels compared to baseline in the analyzed subset of enrolled neonates (SO-IVFE: 9.78 ± 9.72 vs. 19.46 ± 12.24 pg/mL, *p* = 0.000; MCT/ω-3 PUFAs-IVFE: 10.16 ± 9.92 vs. 17.98 ± 15.46 pg/mL, *p* = 0.001). No significant difference was found between groups post-intervention (*p* = 0.070).

## 4. Discussion

In this double-blind controlled clinical trial, premature neonates were randomly allocated to receive either a mixed intravenous fat emulsion containing MCTs, soybean oil, olive oil, and fish oil (MCT/ω-3 PUFAs-IVFE), or the conventional soybean oil-based IVFE (SO-IVFE), starting on the first or second day after birth. 

The results of our study showed that parenteral treatment with MCT/ω-3 PUFAs-IVFE for 15 days led to significantly elevated plasma total ω-3 PUFAs, EPA, and oleic acid concentrations compared to the SO-IVFE group. Furthermore, after 15 days of parenteral nutrition, significantly lower ω-6/ω-3 PUFAs ratio and ω-6 PUFAs were observed in the intervention versus the control group. The raise of LA and ALA levels were found to be higher in the soybean oil-based IVFE compared to the MCT/ω-3 PUFAs-IVFE. The fat emulsion type, however, did not affect serum IL-6 change.

The high LA and ALA content of pure soybean oil emulsions can induce low blood concentrations of their bioactive LC-PUFA metabolites, especially EPA and DHA, and they may be associated with increased rates of infection and lipid peroxidation, exacerbating oxidative stress [30,31,32]. Compared to treatment with SO-based emulsions, administration of lipid emulsions rich in MCT/LC-PUFAs and low in LA and ALA resulted in similar essential fatty acid content in plasma phospholipids and triacylglycerols of preterm neonates [31]. Moreover, excessive intake of LA may promote a persistent inflammatory state that contributes to progressive hepatocyte damage and/or portal inflammation, leading to cholestasis and fibrosis [31]. Literature suggests that a fish oil-containing lipid emulsion including pure fish oil instead of soybean oil alone may be beneficial in prevention of cholestasis in preterm neonates with parenteral nutrition-associated liver disease/cholestasis [33,34,35,36,37,38]. Moreover, pure SO-containing lipid emulsion contains low amounts of the antioxidant a-tocopherol [39]. The low a-tocopherol content may further enhance deleterious lipid peroxidation of the high parenteral PUFA supply. According to recent randomized controlled trials, the MCT/LC-PUFA lipid emulsions enriched with fish and olive oil attenuates the production of pro-inflammatory cytokines and increases total antioxidant capacity in preterm neonates [29,40,41]. In our study, neonates receiving either MCT/ω-3 PUFAs or SO-based lipid emulsions demonstrated significant reductions in serum IL-6, but no significant difference was found between groups.

There are very few clinical trials that investigated the effects of SO-based IVFE and MCT/ω-3 PUFAs-IVFE on the fatty acid profile of preterm neonates. A randomized control trial by Vlaardingerbroek and co-workers (2014) showed that the concentrations of EPA and DHA in triglycerides and phospholipids were significantly higher in the MCT/ω-3 PUFAs compared to the SO group, on days 6 and 14 of parenteral feeding [42]. Other studies showed that the MCT/ω-3 PUFAs-IVFE leads to a lower ω-6/ω-3 fatty acids ratio [43] and lower AA [44] than the soybean oil emulsion. 

To our knowledge, this is the first study showing a favorable effect towards plasma oleic acid concentration after the administration of MCT/ω-3 PUFAs-IVFE, compared to SO-IVFE. In a previously published study conducted by our research team, we observed a rise of plasma oleic acid in the MCT/ω-3 PUFAs group, but no difference was evident between the two groups post-intervention [29]. Another asset of the present study was the detection and quantification of numerous plasma fatty acids after the administration of both MCT/ω-3 PUFAs and SO-IVFE. 

Nevertheless, our study has some limitations. Primarily, the sample size of the study is relatively small due to low availability of preterm neonates needing parenteral nutrition for 15 days. The amount of blood that could be obtained from preterm neonates was limited; consequently, it was impossible to measure serum IL-6 levels in the overall study population or to assess more inflammatory mediators that would probably strengthen our conclusions. The effects of MCT/ω-3 PUFA-enriched IVFE on a combination of serum inflammatory mediators and fatty acid profiles, as well as the associations with nutritional status, morbidity, and long-term outcomes, should be further investigated, in order to determine the safety and the efficacy of this type of IVFE.

## 5. Conclusions

The present study suggests that administration of MCT/ω-3 PUFA-enriched IVFE in preterm neonates is associated with fatty acid profiles consistent with attenuated inflammatory response. The results indicate that the administration of MCT/ω-3 PUFA-IVFE may be beneficial in preterm neonates (for whom parenteral nutrition is necessary) and who are at risk of developing ω-3 PUFAs deficiency. Studies including greater sample sizes and more data such as long-term outcome parameters are of paramount importance in order to determine the efficacy and safety of MCT/ω-3 PUFAs-IVFE administration on preterm neonates.

## Figures and Tables

**Table 1:** Group characteristics at baseline (day 0).

Parameters	*n*	Control Group (SO–IVFE)	*n*	Intervention Group (MCT/ω-3 PUFA-IVFE)	*p*
*N*	46	-	46	-	-
Female	19	-	19	-	-
Male	27	-	27	-	-
Gestational age (weeks)	46	29.76 ± 2.17	46	29.10 ± 1.83	0.126
Birth weight (g)	46	1223.04 ± 215.42	46	1222.74 ± 211.90	0.995
Serum IL-6 (pg/mL)	29	19.46 ± 12.24	30	17.98 ± 15.46	0.383
Fatty acids (% *w*/*w*)					
C14:0	45	0.65 ± 0.16	46	0.68 ± 0.11	0.350
C15:0	40	0.26 ± 0.13	45	0.25 ± 0.17	0.142
C16:0	46	26.52 ± 2.46	46	25.65 ± 2.08	0.070
C16:1 ω-9	46	0.76 ± 0.20	46	0.79 ± 0.19	0.507
C16:1 ω-7	46	3.38 ± 1.17	46	3.47 ± 1.14	0.888
C17:0	46	0.25 ± 0.10	46	0.26 ± 0.11	0.592
C17:1	41	0.21 ± 0.21	45	0.19 ± 0.24	0.343
C18:0	46	9.93 ± 1.92	46	9.60 ± 1.90	0.323
C18:1 ω-9	46	19.75 ± 3.61	46	20.36 ± 3.03	0.385
C18:1 ω-7	46	2.75 ± 0.60	46	2.82 ± 0.58	0.579
C18:2 ω-6	46	8.73 ± 4.90	46	8.51 ± 2.96	0.717
C18:3 ω-6	45	0.43 ± 0.22	46	0.42 ± 0.23	0.533
C18:3 ω-3	40	0.33 ± 0.55	46	0.33 ± 0.26	0.134
C20:0	46	0.45 ± 0.15	46	0.43 ± 0.17	0.520
C20:1 ω-9	40	0.16 ± 0.15	45	0.19 ± 0.18	0.251
C20:2 ω-6	46	0.49 ± 0.34	46	0.46 ± 0.33	0.614
C20:4 ω-6	46	10.36 ± 2.73	46	9.79 ± 2.81	0.329
C20:5 ω-3	46	0.24 ± 0.25	46	0.30 ± 0.50	0.529
C23:0	40	0.30 ± 0.30	44	0.48 ± 0.50	0.144
C24:0	46	0.62 ± 0.12	46	0.57 ± 0.14	0.090
C22:4 ω-6	45	0.35 ± 0.11	46	0.32 ± 0.09	0.130
C22:5 ω-6	45	0.29 ± 0.23	46	0.32 ± 0.34	0.640
C24:1 ω-9	46	1.41 ± 0.39	46	1.49 ± 0.35	0.303
C22:6 ω-3	46	3.24 ± 3.67	46	3.22 ± 3.28	0.970
SFA	46	39.42 ± 4.60	46	38.70 ± 3.94	0.420
MUFA	46	28.81 ± 4.22	46	30.00 ± 4.20	0.179
PUFA	46	25.97 ± 4.64	46	25.45 ± 3.16	0.526
ω-6	46	23.04 ± 4.57	46	21.78 ± 3.08	0.124
ω-3	46	2.94 ± 0.78	46	3.22 ± 0.80	0.120
ω-6/ω-3 ratio	46	7.72 ± 2.31	46	7.28 ± 2.47	0.226

SO, soybean; IVFE, intravenous fat emulsion; MCT, medium-chain triglycerides; PUFAs, polyunsaturated fatty acids; IL-6, interleukin-6; SFAs, saturated fatty acids; MUFAs, monounsaturated fatty acids. Data are expressed as *n* or mean values ± standard deviation of mean (SD). *p* stands for the difference between the control and the intervention group at baseline analyzed by independent sample t-test or the Mann–Whitney test, where applicable. Difference was considered significant at *p* < 0.05.

**Table 2:** Changes in dietary energy intake at baseline (day 0) and at the endpoint of intervention (day 15) in the SO-IVFE group and the MCT/ω-3 PUFAs-IVFE group.

Dietary Intake	Group	Day 0	Day 15	*p*	* *p*
PN energy, kcal/kg/day	Intervention (*n*=46)	51.69 ± 17.03	54.59 ± 28.43	0.591	0.551
	Control (*n* = 46)	51.47 ± 16.26	58.22 ± 23.11	0.086	
Milk energy, kcal/kg/day	Intervention (*n* = 46)	0.00 ± 0.00	48.09 ± 34.32	**0.000**	0.693
	Control (*n* = 46)	0.00 ± 0.00	43.85 ± 34.16	**0.000**	
Total energy, kcal/kg/day	Intervention (*n* = 46)	51.69 ± 17.03	102.69 ± 9.46	**0.000**	0.944
	Control (*n* = 46)	51.47 ± 16.26	101.59 ± 18.30	**0.000**	

SO, soybean; IVFE, intravenous fat emulsion; MCT, medium-chain triglycerides; PUFAs, polyunsaturated fatty acids; PN, parenteral nutrition. Data are expressed as N or mean values ± standard deviation of mean (SD). *p* stands for the difference between the control and the intervention group at baseline analyzed by independent sample t test or the Mann–Whitney test, where applicable; difference was considered significant at *p* < 0.05. * *p* comparison with the baseline values by the paired samples t-test or the Wilcoxon test, where applicable; difference was considered significant at *p* < 0.05; significant *p* values are marked bold in the table.

**Table 3:** Differences in serum fatty acid profile at baseline (day 0) and at the endpoint of intervention (day 15).

Fatty Acids (% *w*/*w*)	*n*	Control Group (SO-IVFE)			*n*	Intervention Group (MCT/ω-3 PUFAs-IVFE)			* *p*
		Day 0	Day 15	*p*		Day 0	Day 15	*p*	
C14:0	45	0.65 ± 0.16	0.97 ± 0.45	**0.000**	46	0.68 ± 0.11	0.86 ± 0.28	**0.001**	0.128
C15:0	40	0.27 ± 0.13	0.17 ± 0.10	**0.000**	45	0.25 ± 0.17	0.17 ± 0.10	**0.000**	0.641
C16:0	46	26.52 ± 2.46	23.87 ± 2.31	**0.000**	46	25.65 ± 2.08	23.60 ± 2.30	**0.000**	0.265
C16:1 ω-9	46	0.76 ± 0.21	0.50 ± 0.14	**0.000**	46	0.79 ± 0.19	0.52 ± 0.11	**0.000**	0.944
C16:1 ω-7	46	3.38 ± 1.17	3.22 ± 1.61	0.175	46	3.47 ± 1.14	3.08 ± 1.12	0.052	0.901
C17:0	46	0.25 ± 0.10	0.20 ± 0.08	**0.000**	46	0.26 ± 0.11	0.19 ± 0.05	**0.000**	0.650
C17:1	41	0.21 ± 0.21	0.17 ± 0.14	0.083	44	0.19 ± 0.24	0.11 ± 0.11	**0.013**	0.703
C18:0	46	9.93 ± 1.92	9.32 ± 1.79	**0.014**	46	9.60 ± 1.90	9.29 ± 1.88	0.289	0.337
C18:1 ω-9	46	19.75 ± 3.61	19.84 ± 2.52	0.933	46	20.36 ± 3.03	23.02 ± 2.86	**0.000**	**0.003**
C18:1 ω-7	46	2.75 ± 0.60	2.29 ± 0.47	**0.000**	46	2.82 ± 0.58	2.21 ± 0.30	**0.000**	0.266
C18:2 ω-6	46	8.73 ± 4.90	17.98 ± 4.35	**0.000**	46	8.51 ± 2.96	16.07 ± 2.70	**0.000**	**0.006**
C18:3 ω-6	45	0.43 ± 0.22	0.57 ± 0.32	**0.040**	46	0.42 ± 0.23	0.44 ± 0.17	0.375	0.231
C18:3 ω-3	40	0.33 ± 0.55	0.56 ± 0.39	**0.000**	46	0.33 ± 0.26	0.38 ± 0.19	**0.030**	**0.006**
C20:0	46	0.45 ± 0.15	0.36 ± 0.17	**0.001**	46	0.43 ± 0.17	0.43 ± 0.26	0.850	0.059
C20:1 ω-9	40	0.15 ± 0.14	0.16 ± 0.10	0.512	45	0.19 ± 0.18	0.18 ± 0.14	0.350	0.406
C20:2 ω-6	46	0.49 ± 0.34	0.43 ± 0.23	0.288	46	0.46 ± 0.33	0.36 ± 0.20	**0.029**	0.417
C20:4 ω-6	46	10.36 ± 2.73	6.46 ± 1.91	**0.000**	46	9.79 ± 2.81	5.16 ± 1.45	**0.000**	0.204
C20:5 ω-3	46	0.24 ± 0.25	0.39 ± 0.26	**0.001**	46	0.30 ± 0.50	0.91 ± 0.58	**0.000**	**0.000**
C23:0	40	0.28 ± 0.29	0.29 ± 0.33	0.421	44	0.48 ± 0.50	0.33 ± 0.35	0.319	0.053
C24:0	46	0.62 ± 0.12	0.50 ± 0.19	**0.001**	46	0.57 ± 0.14	0.44 ± 0.11	**0.000**	0.585
C22:4 ω-6	45	0.32 ± 0.10	0.24 ± 0.11	**0.000**	46	0.35 ± 0.11	0.23 ± 0.15	**0.000**	**0.004**
C22:5 ω-6	45	0.30 ± 0.24	0.18 ± 0.23	**0.000**	46	0.32 ± 0.34	0.18 ± 0.29	**0.000**	0.927
C24:1 ω-9	46	1.41 ± 0.39	1.13 ± 0.31	**0.000**	46	1.49 ± 0.35	1.34 ± 0.28	**0.001**	0.088
C22:6 ω-3	46	3.24 ± 3.67	2.28 ± 2.35	**0.000**	46	3.22 ± 3.28	2.45 ± 2.05	**0.001**	0.204
SFA	46	39.42 ± 4.60	36.28 ± 4.60	**0.000**	46	38.70 ± 3.94	36.29 ± 4.76	**0.000**	0.349
MUFA	46	28.81 ± 4.22	28.12 ± 4.14	0.390	46	30.00 ± 4.20	30.39 ± 3.29	0.575	0.308
PUFA	46	25.97 ± 4.64	30.70 ± 3.64	**0.000**	46	25.45 ± 3.16	28.45 ± 3.11	**0.000**	0.099
ω-6	46	23.04 ± 4.57	28.03 ± 3.55	**0.000**	46	21.78 ± 3.08	24.51 ± 3.03	**0.000**	**0.023**
ω-3	46	2.94 ± 0.78	2.70 ± 0.98	0.131	46	3.22 ± 0.80	3.61 ± 1.51	0.070	**0.031**
ω-6/ω-3 ratio	46	7.72 ± 2.31	12.66 ± 5.20	**0.000**	46	7.28 ± 2.47	8.07 ± 4.58	0.202	**0.001**

SO, soybean; IVFE, intravenous fat emulsion; MCT, medium-chain triglycerides; PUFAs, polyunsaturated fatty acids; SFAs, saturated fatty acids; MUFAs, monounsaturated fatty acids. Data are expressed as mean values ± standard deviation of mean (SD). *p* values: comparison with the baseline values by the paired samples *t* test or the Wilcoxon test, where applicable; difference was considered significant at *p* < 0.05. * *p* values: significant differences in changes at endpoint between the two groups applying the independent samples t-test or the Mann–Whitney test, where applicable; difference was considered significant at *p* < 0.05; significant *p* values are marked bold in the table.

## Supplementary Materials

The following are available online at https://www.mdpi.com/2072-6643/12/11/3526/s1, Table S1: Composition of the MCT/ω-3 PUFAs- and the SO-IVFE, Table S2: Nutrient and mineral intake via enteral and parenteral routes in the SO-IVFE and the MCT/ω-3 PUFAs group at the endpoint of intervention (day 15).
