# Supplementary material for: Administration of an Intravenous Fat Emulsion Enriched with Medium-Chain Triglyceride/ω-3 Fatty Acids is Beneficial Towards Anti-Inflammatory Related Fatty Acid Profile in Preterm Neonates: A Randomized, Double-Blind Clinical Trial
Source: Nutrients. 2020 Nov 16;12(11):3526. doi: 10.3390/nu12113526 (PMC7698253; doi:10.3390/nu12113526)
Supplement: Supplementary file 1 [file nutrients-12-03526-s001.pdf]

Table S1. Composition of the MCT/ω-3 PUFAs- and the SO-IVFE

**Fat emulsion**	**MCT/ω-3 PUFAs-IVFE (Smoflipid)**	**SO-IVFE (Intralipid)**
**Oil source (%)**		
**Soybean**	30	100
**Coconut (MCT)**	30	-
**Olive**	25	-
**Fish**	15	-
**Fatty acids (wt%)**		
**MCTs**		
**C6:0 (caproic acid)**	trace	-
**C8:0 (caprylic acid)**	17	-
**C10:0 (capric acid)**	12	-
**C12:0 (lauric acid)**	0.2	-
**LC-triaglycerols**		
**C14:0 (myristic acid)**	1	0.2
**C16:0 (palmitic acid)**	9	11
**C16:1 ω-7 (palmitoleic acid)**	2	-
**C18:0 (stearic acid)**	3	4
**C18:1 ω-9 (oleic acid)**	29	24
**ω-6 LC-triaglycerols**		
**C18:2 ω-6 (linoleic acid)**	19	53
**C20:4 ω-6 (arachidonic acid)**	0.5	-
**ω-3 LC-triaglycerols**		
**C18:3 ω-3 (α-linolenic acid)**	2	8
**C20:5 ω-3 (eicosapentaenoic acid)**	3	-
**C22:6 ω-3 (docosahexaenoic acid)**	2	-
**Phytosterols (mg/L)**	47.6	348±33
**α-tocopherol (mg/L)**	200	38

SO, soybean; IVFE, intravenous fat emulsion; MCT, medium-chain triglycerides; PUFAs, polyunsaturated fatty acids; LC, long-chain. The manufacturer Fresenius Kabi provided composition data of fat emulsions.

Table S2. Nutrient and mineral intake via enteral and parenteral routes in the SO-IVFE and the MCT/ω-3 PUFAs group at the endpoint of intervention (day 15)

Dietary intake	Group	Mean values ±SD	*p*
**Protein, g/kg/d**	Intervention (N=46)	3.1 ± 0.63	0.862
	Control (N=46)	3.2 ± 0.32	
**Fat, g/kg/d**	Intervention (N=46)	3.0 ± 0.70	0.949
	Control (N=46)	3.0 ± 0.41	
**Glucose, g/kg/d**	Intervention (N=46)	13.0 ± 2.6	0.496
	Control (N=46)	13.3 ± 1.4	
**Sodium, meq/kg/d**	Intervention (N=46)	2.8 ± 1.3	0.129
	Control (N=46)	2.9 ± 1.3	
**Potassium, meq/kg/d**	Intervention (N=46)	2.0 ± 0.3	0.316
	Control (N=46)	2.0 ± 0.4	
**Calcium, meq/kg/d**	Intervention (N=46)	2.0 ± 0.6	0.057
	Control (N=46)	2.1 ± 0.5	
**Magnesium, meq/kg/d**	Intervention (N=46)	0.76 ± 0.1	0.676
	Control (N=46)	0.77 ± 0.1	
**Phosphate, meq/kg/d**	Intervention (N=46)	1.2 ± 0.2	0.151
	Control (N=46)	1.3 ± 0.3	

SO, soybean; IVFE, intravenous fat emulsion; MCT, medium-chain triglycerides; PUFAs, polyunsaturated fatty acids. Data are expressed as mean values ± standard deviation of mean (SD). *p* stands for the difference between the control and the intervention group analyzed by independent sample t test or the Mann-Whitney test, where applicable; difference was considered significant at $p < 0.05$.
